# Speed-accuracy tradeoffs in human speech production

**DOI:** 10.1371/journal.pone.0202180

**Published:** 2018-09-07

**Authors:** Adam C. Lammert, Christine H. Shadle, Shrikanth S. Narayanan, Thomas F. Quatieri

**Affiliations:** 1 MIT Lincoln Laboratory, Lexington, Massachusetts, United States of America; 2 Haskins Laboratories, New Haven, Connecticut, United States of America; 3 Signal Analysis and Interpretation Laboratory, Los Angeles, California, United States of America; University of Kent, UNITED KINGDOM

## Abstract

Speech motor actions are performed quickly, while simultaneously maintaining a high degree of accuracy. Are speed and accuracy in conflict during speech production? Speed-accuracy tradeoffs have been shown in many domains of human motor action, but have not been directly examined in the domain of speech production. The present work seeks evidence for Fitts’ law, a rigorous formulation of this fundamental tradeoff, in speech articulation kinematics by analyzing USC-TIMIT, a real-time magnetic resonance imaging data set of speech production. A theoretical framework for considering Fitts’ law with respect to models of speech motor control is elucidated. Methodological challenges in seeking relationships consistent with Fitts’ law are addressed, including the operational definitions and measurement of key variables in real-time MRI data. Results suggest the presence of speed-accuracy tradeoffs for certain types of speech production actions, with wide variability across syllable position, and substantial variability also across subjects. Coda consonant targets immediately following the syllabic nucleus show the strongest evidence of this tradeoff, with correlations as high as 0.72 between speed and accuracy. A discussion is provided concerning the potentially limited applicability of Fitts’ law in the context of speech production, as well as the theoretical context for interpreting the results.

## Introduction

The present work applies certain influential ideas of Paul Fitts [1] in the domain of speech production, specifically his formulation of so-called speed-accuracy tradeoffs in human motor action. Fitts was primarily concerned with quantifying the capacity of the human motor system to perform motor actions. One important outcome of that work was a rigorous formulation of perhaps the most robust and widely replicated laws of human motor action: for discrete, targeted actions, the time taken to complete a movement displays a linear relationship with task difficulty, where difficulty is a function of movement distance and the tolerable error in reaching the target. This law is typically described as a speed-accuracy relationship, given the intuitive notion that movement time and speed are quantities that are closely, if inversely, related, and that tolerable error is the reciprocal of accuracy. This now well-known relationship has subsequently been referred to as *Fitts’ law*, and has been used widely to model speed-accuracy tradeoffs in a variety of human movement domains. Example application domains include manual pointing and reaching (as in Fitts’ original study), targeted foot movements [2], balance and posture [3], and computer device interaction [4]. Fitts’ law has also been applied to ballistic movements, including eye saccades [5], although there is meaningful debate over whether movements that do not rely heavily on feedback are subject to the same law [6–8].

It is not well established whether Fitts’ law is obeyed by speech motor actions. Conceptualizing speech actions as discrete motor actions, there is evidence that speech articulation obeys tradeoffs among related metrics of speed, distance and curvature [9–11]. However, Fitts’ law has not been directly examined in the context of speech production. Motor actions associated with speech production appear to be performed quickly, while simultaneously maintaining a high degree of accuracy. The presence of speed-accuracy tradeoffs would imply that this situation might not be possible, if speed and accuracy are in conflict during speech production. Moreover, in speech production, there are potentially multiple domains in which accuracy is demanded, including articulatory, acoustic, prosodic, and communicative, with all of these demands being potentially simultaneous and overlapping. The present work focuses on the kinematics of “reaching” for maximal articulatory targets in speech, because studies from other domains of human movement have most commonly found clear speed-accuracy tradeoffs in the kinematics of discrete, targeted actions.

Speed-accuracy tradeoffs can provide a window into the control mechanisms of directed movements. While it is possible that biomechanical constraints exist that give rise to such tradeoffs, there is also good reason to believe that they are the result of properties of planning and control. It can be shown that Fitts’ law is consistent with traditional models of feedback-driven motor control [12]. Moreover, it is closely related to models of neural dynamics of movement trajectory formation [13]. If it is true that control mechanisms bring about speed-accuracy tradeoffs, it implies that changes in timing can be used to assess demands in accuracy and, conversely, that changes in accuracy can be partially attributed to speaking rate demand.

The presence of Fitts-type tradeoffs in speech production would help to explain a variety of observed phenomena. Hardcastle [14] argued that speech motor actions vary in terms of their difficulty, where difficulty (i.e., what Hardcastle calls *complexity*) is defined in terms of both the number of articulatory variables that are recruited over the course of that action, more relevant to the present discussion, and in terms of the precision required for each of those variables. The issue of articulatory precision is entirely compatible with Fitts’ law. Hardcastle proceeds to relate differences in difficulty to aspects of articulatory timing. In the process of arguing that fricatives require more precision than stop consonants, for instance, he makes direct reference to speed-accuracy tradeoffs: “One of the possible effects of this greater precision is that the articulators involved in the production of a fricative might move more slowly than for the production of a stop.” Hardcastle notes that, because more time would be required to execute a more difficult fricative articulation, this may help to explain why vowels are often lengthened in advance of fricatives, a suggestion originally made by MacNeilage [15]. Relatedly, because more time would be required for the tongue to travel a longer distance, this may help explain why lower vowels tend to be longer than higher vowels [16]. The tradeoff Hardcastle discusses is also a possible explanation for the observation that, to a broad first approximation, fricatives have longer durations than stops [17].

The purpose of this paper is three-fold. The primary goal is to analyze speech articulation using a large database of real-time magnetic resonance (rtMRI) data, in order to assess whether articulatory kinematics conform to Fitts’ law. A second, associated goal is to address the methodological challenges inherent in performing Fitts-style analysis on rtMRI data of speech production. Methodological challenges include segmenting continuous speech into specific motor tasks, defining key variables of Fitts’ law in the domain of speech articulation, and deciding how to operationalize these definitions and extract related measures from complex and high-dimensional rtMRI data. Finally, a third goal is to present a novel mathematical argument for Fitts’ law in speech production, and make a theoretical argument for why one would expect to observe behavior consistent with the law. Section 2 gives a brief introduction to the concepts and mathematics behind Fitts’ law, and presents an argument for Fitts’ law in speech production. Section 3 describes the data used in the present study, and the necessary pre-processing for the task being considered. Section 4 explains the present approach to applying Fitts’ law in the domain of speech production data. The results of applying the proposed methodology to rtMRI data, and a discussion of the results in terms of the goals of the paper, are given in Section 5.

Lammert et al. [18] have previously reported on an initial effort to meet some of the goals of the present work by analyzing portions of the USC-TIMIT database and forming necessary elements of the data analysis. This paper constitutes a substantial expansion of that work, providing a more extensive and deeper analysis of a larger number of subjects, as well as a better developed framework for considering speed-accuracy trade offs in a speech production context. In particular, the present work provides (1) an analysis of six additional subjects, altogether comprising the entirety of the real-time data from the USC-TIMIT database, (2) a more detailed look at speed-accuracy relationships in speech tasks of different varieties, specifically tasks situated in different parts of the syllable, and (3) elucidation of a theoretical framework for considering Fitts’ law in the domain of speech production, and its mathematical connection to prominent models of speech motor control and neural control of movement. None of the results reported in the present manuscript have been previously reported in the published literature.

## Background

Fitts’ law can be stated precisely in mathematical terms. It has deep connections with several prominent frameworks of directed human motor control. This section is intended to provide an overview of Fitts’ law, including the mathematical statement thereof, as well as connections to the Task Dynamics control framework [19, 20], and the VITE model of neural control of directed human movement [13].

### Statement of Fitts’ law

Without reference to any specific motor domain, Fitts’ law can be described in purely abstract terms. A given motor task can be said to have a spatial *target* which is the end state associated with the desired action, as well as an *initial position* which is the state from which the action begins. The initial position can also be thought of as the *context* in which the task takes place. Other key variables of the task can then be defined, including the *distance* to the target from the initial position, and the *width* of the target in terms of its spatial extent. Longer distances are assumed to make a task more difficult, whereas larger widths are assumed to make the task easier because width represents the tolerable error in reaching the target.

The ratio of the distance to the target, D, and its width, W, are then associated with the *index of difficulty* (ID) in the following way:

$$\begin{array}{c} ID={\text{log}}_{2}(\frac{2D}{W}) \end{array}$$ (1)

The reciprocal ratio *W*/*D* constitutes one definition of the accuracy of the performance of a task. Taking the base-2 logarithm of this accuracy measure, then, gives the ID units that can be interpreted as bits, inspired by Claude Shannon’s information theory [21]. The ID, having encapsulated a notion of accuracy of action, should then be related to the *movement time* (MT) associated with a given task, under the hypothesis that a tradeoff exists between speed and accuracy of the performance of that task. This relationship, Fitts’ law, is commonly formulated as a simple, linear one:

$$\begin{array}{c} MT=a\cdot ID+b, \end{array}$$ (2)

where *a* and *b* are constants, the values of which depend on the task and characteristics of control. Fitts’ law has been derived in various ways since the original formulation [13, 22, 23].

The distance associated with a task has historically been defined as the Euclidean distance between the initial position and the target. Width, on the other hand, has historically been the subject of debate, which has resulted in several different definitions for this variable. Fitts’ original experiments included targets for which the (variable) spatial extent was firmly and sharply defined. Some other experimental setups have included only a point target, with width being defined in terms of dispersion relative to that target. In the domain of speech production, an additional layer of complication stems from a lack of consensus regarding how articulatory targets should be defined, or indeed whether an *articulatory* (as opposed to acoustic) target exists at all. The present work assumes that articulatory targets do exist, following the specific definition explained below.

It is worth noting certain subtleties with regard to the interpretation of Fitts’ law as an expression of a speed-accuracy trade off. Much of the literature related to Fitts’ law interprets the law as such a trade off, either implicitly or explicitly. For example, Fitts et al. [24] discuss the variables MT and W as representing speed and the reciprocal of accuracy, respectively. The law, under this interpretation of the variables, is therefore an expression of a speed-accuracy trade off, with that additional caveat that accuracy must always be considered relative to D. This interpretation of Fitts’ law assumes that “speed” is the reciprocal of MT—essentially an expression of the speed of completion of the task—rather than articulator speed, as in the classical-mechanical sense of |*D*/*MT*|. A classical definition of the speed-accuracy trade off might be *W*_1_ = *cD*/*MT*, stating that *W*_1_ is proportional to articulatory velocity, given some coefficient *c*. This classical definition is not exactly the same as Fitts’ law, but the two can be related by rewriting Eq 2 as: *MT* = log_2_(*cD*) − log_2_(*W*_2_), implying that *W*_2_ = *cD*/2^*MT*^ (ignoring coefficients, for simplicity, and substituting *c* for the value 2). The quantity *cD*/2^*MT*^ is still not the classical definition of speed, but it similarly decreases monotonically with *MT*, and the quantities *W*_1_ and *W*_2_ from the Fitts’ and classical definitions can be related by a multiplicative factor, *W*_1_ = *ηW*_2_, where *η* = 2^*MT*^/*MT*.

### Theoretical framework

Fitts’ law has substantial mathematical connections with the dynamical systems view of coordination and control of human movement (e.g., [25, 26]). This section attempts to elucidate those connections, and to provide a novel argument for expecting behavior consistent with Fitts’ law in speech production on the basis of prominent theories of speech motor control and neural dynamics. Within the dynamical systems perspective, one representative body of work that has had an impact on modeling and explaining speech articulation is that of Task Dynamics [19, 20]. Task Dynamics constitutes a control system that allows for the description and achievement of directed actions in a relatively high-level *task space*, as opposed to the relatively low-level *articulator space*, defined by variables of mobility such as muscle activations. An example of a task space for a manual reaching task would be three-dimensional Cartesian space, as opposed to the articulatory space of joint angles at the shoulder, elbow and wrist. Task space for a speech production action could be the space defined by the first three formant frequencies, or the space defined by vocal tract constriction degree and location, as in Articulatory Phonology [27]. These high-level spaces are the natural spaces in which to define the goals of directed action, and Task Dynamics defines a rigorous framework in which motor commands can be generated in articulator space toward the completion of movements in task space.

**Fig 1:**
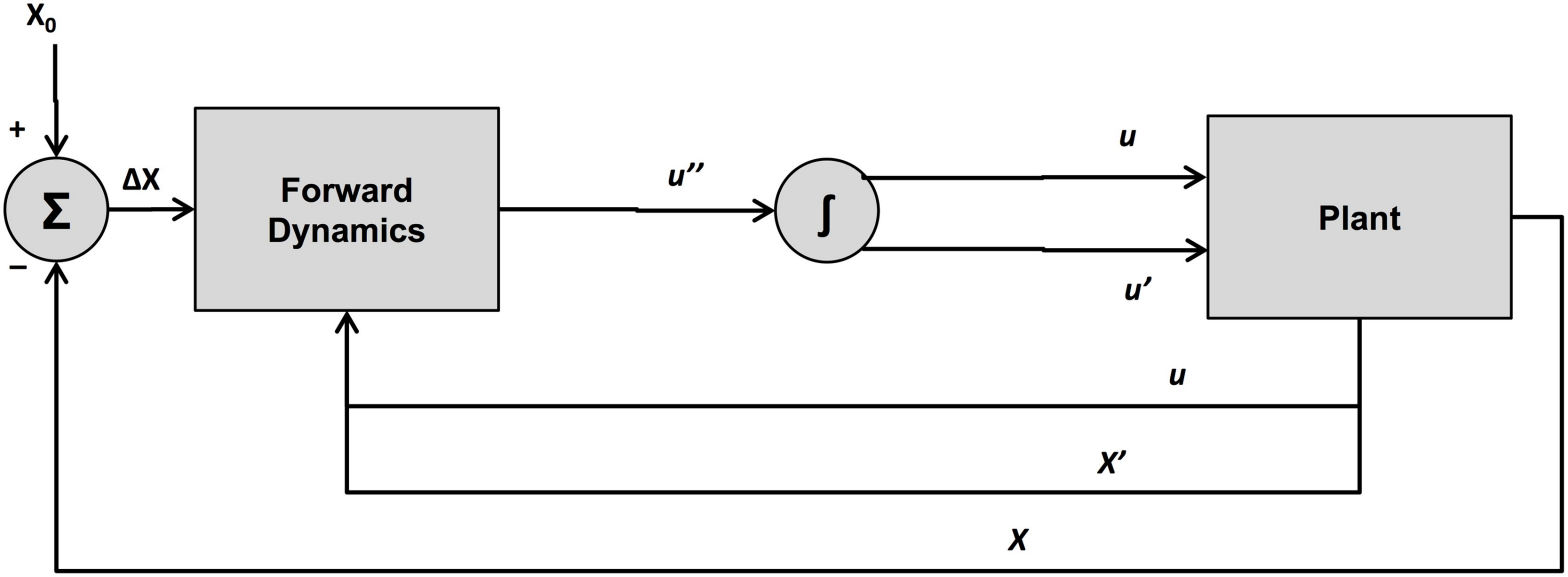
Schematic representation of the Task Dynamics framework. The variable *X* is the displacement of the controlled variable in task space and *X*_0_ is the target. The Forward Dynamics component implements a second-order dynamical system, conforming to Eq 3, that transforms (via inverse kinematics) the error signal, Δ*X*, into the second derivative of the articulator-space variable *u*. The integrals $\dot{u}$ and *u* function as motor commands to the Plant, or speech production apparatus.

In Task Dynamics, the targets of directed movement are assumed to be points in task space. Those targets are achieved by point-attractor dynamics, governed by 2^*nd*^-order equations of motion consistent with a critically damped harmonic oscillator. the dynamics of which are well understood from classical mechanics. For the sake of simplicity, consider a one-dimensional task space. The equations can be written as follows:

$$\begin{array}{c} \ddot{X}=\frac{-c\dot{X}}{m}-\frac{k(X-X_{0})}{m}, \end{array}$$ (3)

where *X* is the displacement of the controlled variable and *X*_0_ is the target. The forward dynamics take the form of a second-order dynamical system, conforming to Eq 3, that transforms the error signal, Δ*X*, into the second derivative of the articulator-space variable *u*. An overview of the control flow in Task Dynamics is shown in Fig 1. Eq 3 is contained within the box labelled “Forward Dynamics”, which computes the acceleration of *u* from Δ*X* = *X*_0_ − *X*. Note that the low-level articulator variables, *u*, and the relevant kinematic transformations between task and articulator spaces are not discussed in the present context. This is because dynamics in task space only are sufficient to account for Fitts’ law.

Fitts’ law can be seen as a direct consequence of such dynamics. A mathematical connection can be made through an examination of the step response of the system, which corresponds to the sudden appearance of a new target in task space. The relevant quantity then becomes the settling time of the damped harmonic oscillator, that is, the time required for the system to converge within a certain percentage of the final target value, beginning at rest. It is well known from classical mechanics that, in the case of critical damping, the rate of convergence in the step response to a change in target follows a decaying exponential. That is, the displacement of the system at time *t* is *X*_*t*_ = *X*_0_ *e*^−*ω*_0_*ζt*^, in a system where the natural frequency is $\omega_{0}=\sqrt{k/m}$, and the damping ratio is *ζ* = *c*/2*mω*_0_. In the case of critical damping, *ζ* = 1, and $X_{t}=X_{0}e^{t\sqrt{k/m}}$.

Several of these quantities can be related directly to those in the formulation of Fitts’ law. The value *t* can be considered as *MT*, the time at which the system is considered to have settled, or completed its action. Given that the movement takes time *t* to complete, and *X*_*t*_ is the residual displacement of the controlled variable after the action has completed, *X*_*t*_ can be equated with the error tolerance *W*. Furthermore, *X*_0_ is equivalent to the movement distance, *D*, if the movement is considered to begin at *X* = 0. Following from these identities, we can express the step response equation above with a change of variables, as $W=De^{-\sqrt{k/m}MT}$. This can be easily rewritten as:

$$\begin{array}{c} MT=\frac{1}{\sqrt{\frac{k}{m}}}\cdot \ln(\frac{D}{W}), \end{array}$$ (4)

which is already similar to Fitts’ law in form. We can find the conditions under which they are equivalent by setting this new formula for *MT* equal to the one taken from Fitts’ law. Beginning—for the sake of clarity—with a change of logarithm base from the law expressed in Eq 2 (corresponding to a switch of units in ID from *bits* to *nats*), we have:

$$\begin{array}{c} a\cdot \ln(\frac{2D}{W})+b=\frac{1}{\sqrt{\frac{k}{m}}}\ln(\frac{D}{W}) \end{array}$$ (5)

It is easy to show that this equation holds for certain values of *a* and *b*. For instance, assuming that $a=\frac{1}{\sqrt{k/m}}$ (the reciprocal of the natural frequency of oscillation), one can solve to find that $b=\frac{\ln(2)}{\sqrt{k/m}}$. Therefore, Fitts’ law conforms to the predicted kinematic behavior of a damped harmonic oscillator, which is consistent with the behavior of a Task Dynamic control system when acting to achieve a specific movement target.

In addition to the kinematic considerations of the Task Dynamics model, Fitts’ law also has substantial mathematical connections with models of the neural dynamics underlying the dynamical systems view of human motor control. An influential neural-inspired network model for explaining kinematic trajectory formation of directed movement is the VITE model [13]. This model’s predictions are highly consistent with those of the Task Dynamics model, owing to the fact that VITE is a 2^*nd*^-order dynamical system much like Task Dynamics (as pointed out by, e.g., [23]). VITE comprises a network of interacting hypothesized neural populations which generate a movement command, given some target position. The neural populations are configured in order to code distinct quantities that are needed in the generation of the motor command. Among the interacting neural populations, there is (a) a population representing the target position command (TPC), (b) a population representing the present position command (PPC), and (c) a population referred to as the difference vector (DV) population, which represents the difference between the PPC and TPC.

**Fig 2:**
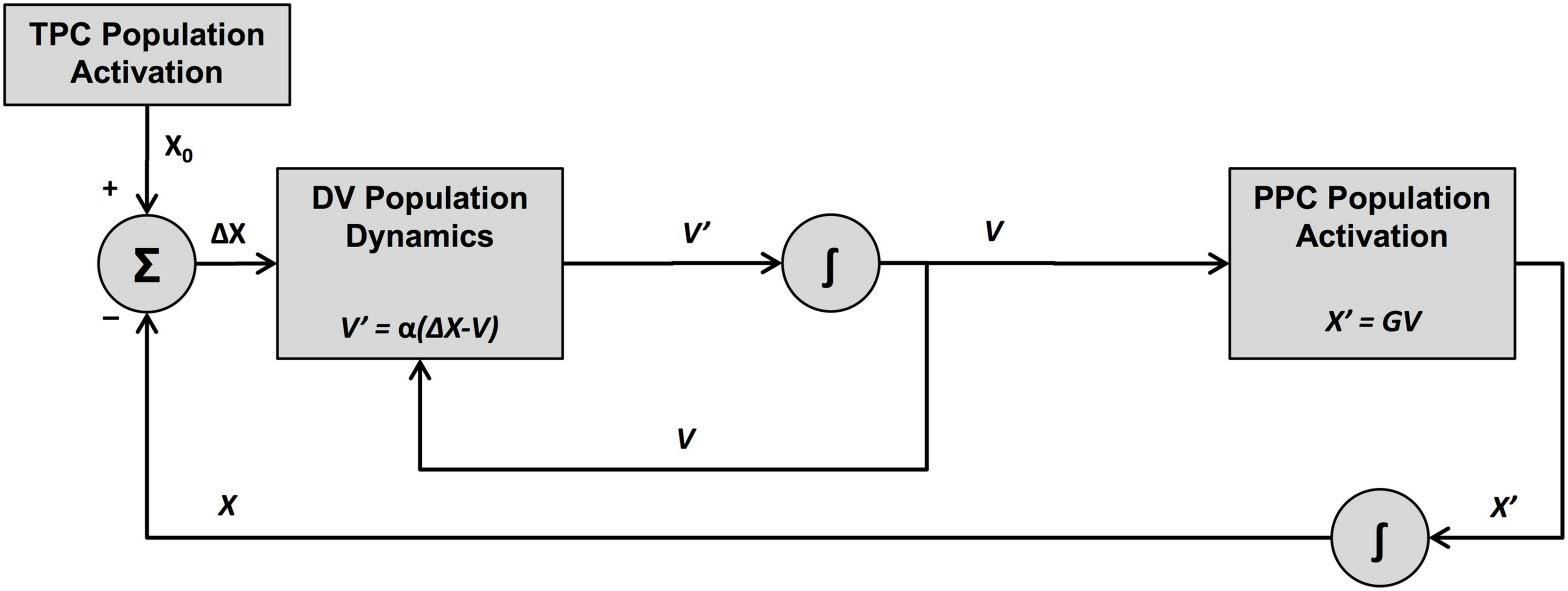
Schematic representation of the VITE neural model [13]. Note the many similarities of this structure to that of Task Dynamics in Fig 1. TPC is a representation of the target position, which produces a target position *X*_0_. The DV population compares the target to the system’s current position, and computes the task-space dynamics of the network. The PPC population integrates the DV population activation into position information. The network dynamics have the form described in Eqs 6 and 7.

The specific structure of VITE’s interacting network is shown in Fig 2. Note the many similarities of this structure to that of Task Dynamics in Fig 1. TPC, as a representation of the target position, produces a target position *X*_0_. The DV population compares the target to the system’s current position, and computes the task-space dynamics of the network. The PPC population, meanwhile, integrates the DV population activation into position information, in analogy to the physical plant in the Task Dynamics control flow. The network dynamics have the following form:

$$\begin{array}{c} \dot{V}=\alpha \left(X_{0}-X-V\right), \end{array}$$ (6)

and

$$\begin{array}{c} \dot{X}=GV \end{array}$$ (7)

where the parameter *α* has been termed the “convergence coefficient” and *G* is the “go” signal, which initiates and sustains movement. These equations also compare easily to the equations of motion for Task Dynamics given above in Eq 3. There are important differences, however. First, all computations are done at the level of tasks, with no mention of the articulator space. Therefore, there is no need for kinematic transformations between task space and articulator space in VITE. Second, the inclusion of *G* has no equivalent in Task Dynamics, where it is assumed (implicitly) that movement toward a target is always active as long as the target exists. This presentation also glosses over a nonlinearity in the original VITE formulation, where V is not allowed to go negative, which was viewed as unimportant in the present context.

As with Task Dynamics, Fitts’ law can be seen as a direct consequence of these neural-inspired dynamics. This can be shown by demonstrating the mathematical relationship between the equations of motion in Eqs 6 and 3. If *G* = 1, then $V=\dot{X}$, allowing Eqs 6 and 7 to be combined into the single equation:

$$\begin{array}{c} \ddot{X}=\alpha \left(X_{0}-X-\dot{X}\right), \end{array}$$ (8)

which is the same as Eq 3, if *α* = −*k*/*m* = −*c*/*m*. Therefore, VITE is consistent with Task Dynamics control, and Fitts’ law can be seen as related to both those models in a general sense, and as a direct consequence of them under the specified conditions and parameters. Note, incidentally, that because the damping coefficient in VITE is fixed at *c* = −*αm*, in order for the system to be critically damped (i.e., $c=2\sqrt{mk}$), as in Task Dynamics, that *m* = *k*/4. Overdamping will occur with *m* > *k*/4, and underdamping with *m* < *k*/4.

### Methodological framework

**Fig 3:**
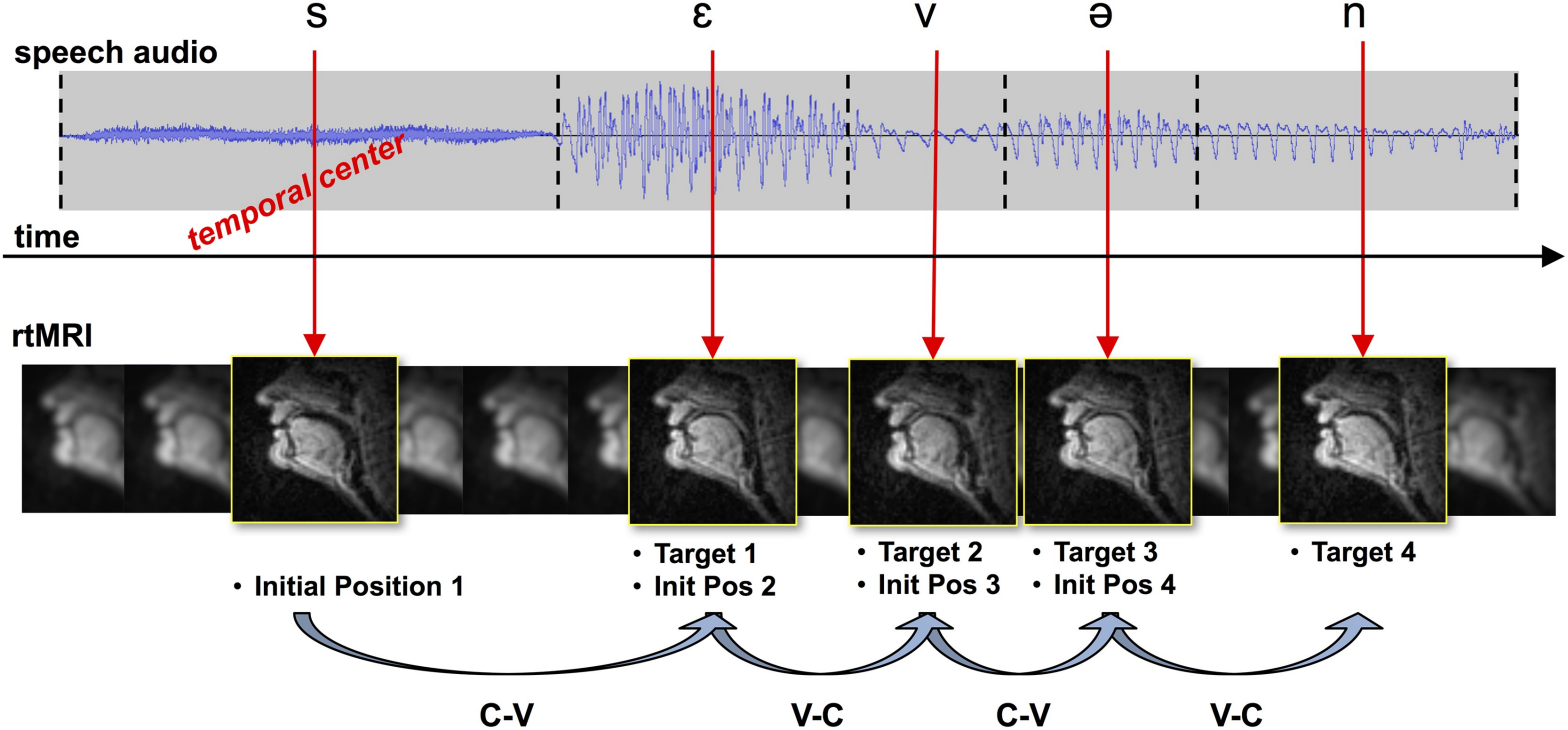
A key concept behind the methodology developed in the present work is that motor tasks in speech articulation can be viewed as a sequence of movements toward and away from target points in articulatory space. Those targets are assumed to be approached and approximated, but not necessarily reached, at the temporal center of each phone interval. The initial position for a given task is assumed to be the target immediately preceding the current one.

**Fig 4:**
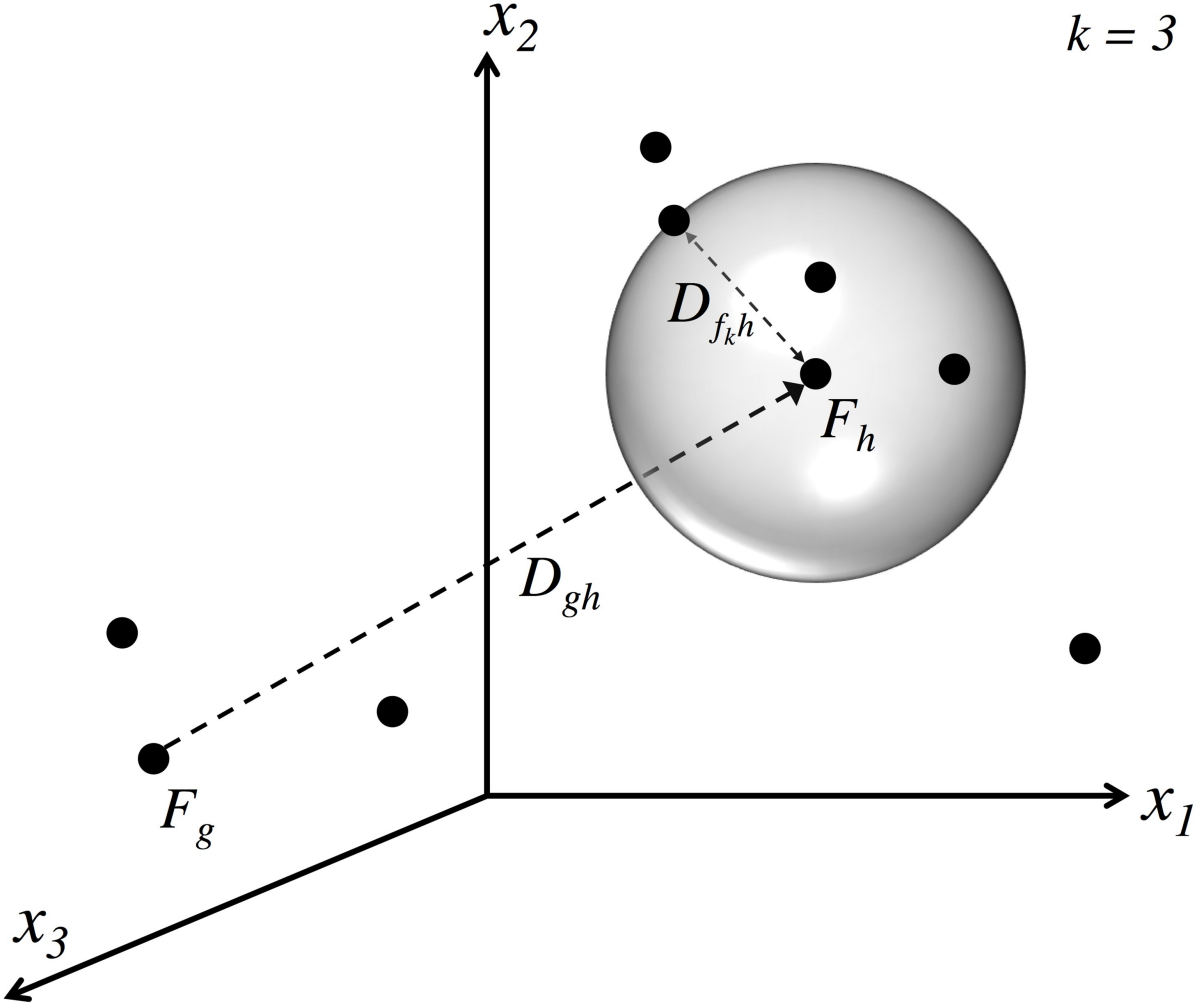
Illustration of the key relationships in calculating ID from articulatory data, with most variable names taken from the text. Target vectors are defined in the high-dimensional articulatory space, represented in the illustration by features *x*_1_, *x*_2_, *x*_3_. In the analysis, this articulatory space is actually composed of *L* total features. The articulatory target vector *F*_*g*_ is the target of the previous movement, and represents the starting point of the current movement. The target of the current movement is *F*_*h*_. The distance to the target is the Euclidean distance between these two vectors. The width around the target is calculated with respect to a hypersphere around the current target, which is used to estimate the density of other target vectors that are not the current one.

To facilitate analysis of speech production data in a way consistent with Fitts’ law, the assumed targets of speech articulation must be operationally defined in both space and time. It is assumed in the present work that each phoneme is associated with a single articulatory target. The initial position for a given task is assumed to be the target of the task immediately preceding the current task. Thus, an utterance of continuous speech can be conceptualized as a sequence of task-related movements away from the previous phoneme target and toward the subsequent target. Each individual task, conceptualized in this way, can be referred to by a diphone representing a context-target task pair. These notions will be defined formally below, and are shown visually in Fig 3. In spatial terms, it is assumed that the target of a given phoneme can be described by a vector in some high-dimensional articulatory space. It is further assumed that a given task-related action comes closest to achieving its target at the temporal center of the associated phone interval. Targets might not be reached during continuous speech for a variety of reasons, including articulatory undershoot, errorful articulation, or inherent tolerance of the controller to some deviation from the target (e.g., due to, perhaps, categorical realization of speech targets). The location of the target vector for a particular task will be operationally approximated by taking the mean of all examples of a given phoneme label. These notions will be defined formally below, and are shown visually in Fig 4.

**Fig 5:**
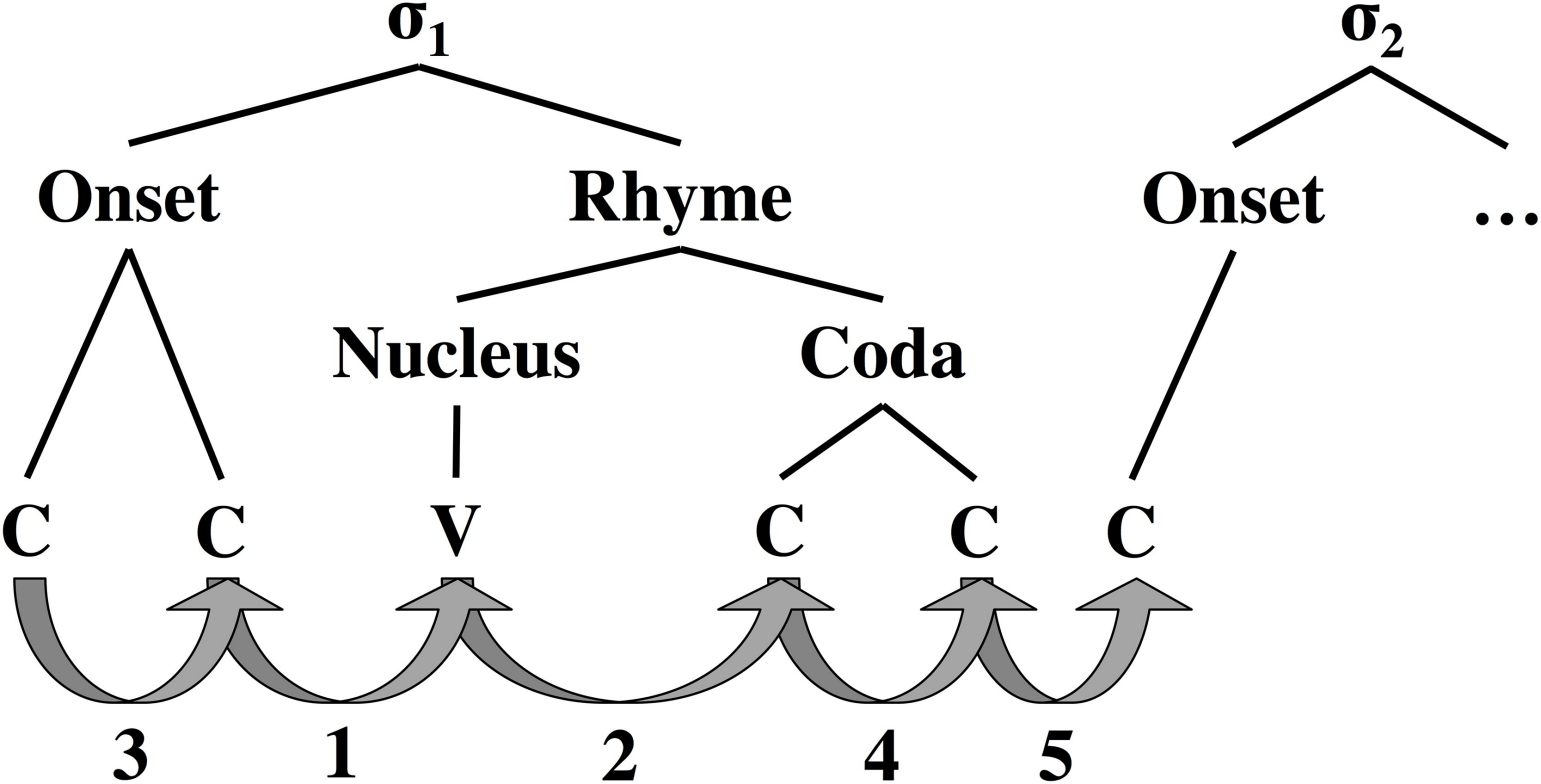
Illustration of the different syllable position-specific task categories used in the present analysis, shown on a traditional, generic syllable structure tree. Categories are numbered outward from the nucleus, and include tasks leading into and out of the nucleus (1 & 2), tasks between consonants in the onset and coda (3 & 4) and tasks leading from one syllable to the next (5).

It has been well-established that the temporal relationship between speech gestures varies as a function of their positions within the syllable [28–30]. Therefore, it was hypothesized that adherence to Fitts’ law might vary depending on the task type, where type was determined by the position of a diphone within the syllable. To facilitate analysis of speech tasks conditioned on syllable position, a syllabification was performed and five categories of interest were defined with respect to syllable structure (see Fig 5):

- Category 1: Onset-Nucleus Task (initial position: final onset consonant; target: syllable nucleus)
- Category 2: Nucleus-Coda Task (initial position: syllable nucleus; target: first coda consonant)
- Category 3: Onset-Onset Task (initial position: onset consonant; target: succeeding onset consonant)
- Category 4: Coda-Coda Task (initial position: coda consonant; target: succeeding coda consonant)
- Category 5: Coda-Onset Task (initial position: final coda consonant; target: first onset consonant of succeeding syllable)

Note that the tasks in category 5 are across syllables, including in some cases across a word boundary, whereas the tasks in categories 1–4 are all within a single syllable. Note also that not every syllable contained tasks from each of these five categories. For instance, syllables conforming to a VCC structure would not contain tasks from categories 1 or 3, and potentially not category 5, depending on the presence of preceding or succeeding syllables.

## Method

### Data, pre-processing & feature extraction

All human subjects research for this study was approved by the University of Southern California Institutional Review Board (University Park IRB). Data used in the present analysis were taken from the USC-TIMIT data set [31], collected by the Speech Production and Articulation kNowledge group at the University of Southern California (the full data set is publicly available online at http://sail.usc.edu/span; a minimal data set used for the present analysis is available on Dryad Digital Repository, doi:10.5061/dryad.5pn163j). USC-TIMIT is a multimodal collection of speech production data from 10 speakers of American English—five male (M) and five female (F)—reading aloud the 460 sentences of the MOCHA-TIMIT corpus [32]. All subjects were used in the present analysis (i.e., M1-5 and F1-5). USC-TIMIT contains data gathered from both midsagittal rtMRI and electromagnetic articulography (EMA), but only the rtMRI data was utilized in the present analysis. The rtMRI data had been reconstructed at an effective frame rate of 23.18 frames/second, with a spatial resolution of 68 by 68 pixels and pixels representing approximately a 2.9*mm*^2^ area. Audio was simultaneously recorded at a sampling frequency of 20 kHz using an MRI-safe optical microphone. MR scanner noise was removed from the audio using an algorithm developed by Bresch [33], and the audio was subsequently used to perform forced phoneme alignment with the SAIL-Align noise-robust alignment tool [34]. For three of the speakers, software-related technical difficulties resulted in a subset of MRI frames going unrecorded in the data, which makes ideal audio-video synchronization impossible. Sentences in which these difficulties arose were discarded. In the end, 346 of the 4600 total sentences were discarded, including 175 from F4 (sentences 286 to 460), 166 from M5 (sentences 295-460) and only five sentences from M3 (sentences 331-335). All 460 sentences were represented in the data for the other seven subjects.

Analysis of speech production kinematics began with a matrix-formatted image sequence, *X* = [*I*_1_*I*_2_*I*_3_…*I*_*n*_]^*T*^, comprising all *n* image frames *I*_*m*_ in the corpus from a single subject. Individual image frames, *I*_*m*_, were in vectorized format, meaning that pixels located at (*i*, *j*) in rectangular *r* by *c* image format were located at *c*(*i* − 1) + *j* in the vector *I*. *I* is of length *rc*. The gray-scale intensity values of each pixel in the image (i.e., each column in *X*) were considered candidate articulatory features [35, 36], subject to the pre-processing and transformations described below. This direct, pixel-wise approach may provide articulatory features that are less interpretable than traditional phonetic descriptions (e.g., tongue height, lip closure, etc.). However, pixel-wise features provide a holistic analysis of the entire midsagittal plane, while simultaneously minimizing assumptions about what information might be important for describing articulation. Pixel-wise analysis is also robust when applied to low-contrast, low spatial-resolution rtMR images [37] because relatively brittle edge-detection/boundary-extraction algorithms are not required. Subjects were analyzed separately, due to concerns about the proper method of combining articulatory features across subjects.

Image vectors underwent an intensity correction procedure to compensate for the reduction in coil sensitivity at increasing spatial distances from the coils themselves, which were positioned a few centimeters in front of the nose and lips, extending laterally and slightly curving around to the sides of the head and neck. Lowered coil sensitivity results in lower mean pixel intensity values and smaller dynamic range for a given pixel location. Intensity correction is used in an attempt to normalize pixel intensity values, so that pixels can be compared and interpreted across all spatial locations in the image plane. A retrospective correction scheme was implemented, incorporating a nonparametric, monotonically increasing estimate of coil sensitivity derived from the pixel values in *X* [36]. Image intensity correction results in a matrix *X*^*c*^ of corrected image vectors.

Candidate articulatory features were removed from consideration if they displayed relatively static values over the image sequence. Features displaying this kind of behavior were assumed to be unrelated to vocal tract action. Approximately 75% of all pixels in the images were representative of, for instance, portions of the spine or the air in front of the face, as determined by visual inspection of the images. A simple low-variance threshold procedure was implemented to remove them from consideration, in which the variance along columns of *X*^*c*^ was calculated. Columns with the lowest variance were eliminated as candidate features. Therefore, the matrix $X_{sub}^{c}$ was formed, which contained only those columns of *X*^*c*^ with variance above the 74^*th*^ percentile across all columns. The matrix $X_{sub}^{c}$ is therefore *n* by *rc*/4 in size.

Only a subset of the *n* data vectors in the matrix $X_{sub}^{c}$ represent vocal tract configurations in close temporal proximity to an articulatory target, using the present operational definition of articulatory targets (described above). To further focus analysis on targeted articulatory actions, the row vectors in $X_{sub}^{c}$ corresponding to the temporal centers of phones were identified and extracted using the results of a forced phoneme alignment to the recorded speech audio. Each phone was assigned a starting boundary *A*_*m*_, and an ending boundary *B*_*m*_, both in seconds. From these, the temporal center of a phone can be calculated as Γ_*m*_ = (*A*_*m*_ + *B*_*m*_)/2, and the corresponding image frame is arg_*m*_ min(Γ_*m*_ − *τ*_*m*_)^2^ for timestamps *τ*_1_, …, *τ*_*n*_ associated with each original image frame. A new matrix *Y* can be formed from this calculation, which is *P* by *rc*/4 in size, where *P* is the total number of phones represented in the image sequence. In the data analyzed here, $P\approx 1.5\dot{1}0^{4}$ articulatory targets.

**Fig 6:**
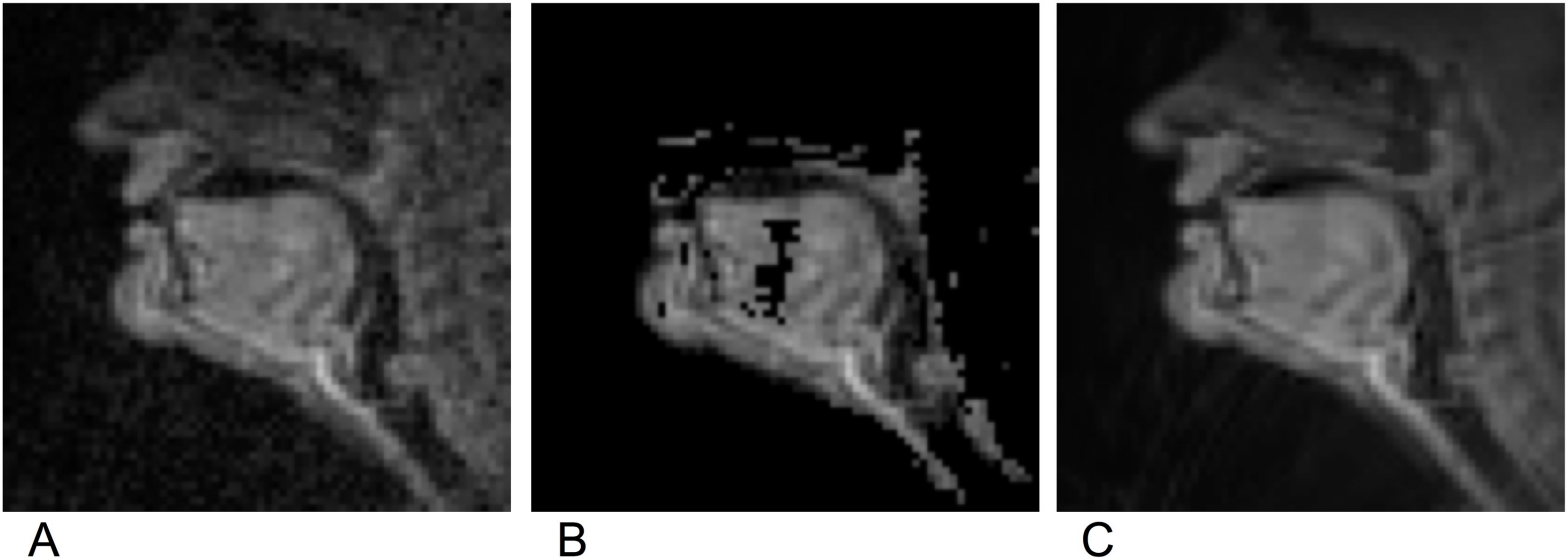
Images illustrating stages in the data pre-processing pipeline for a single vocal tract posture. Shown are (a) an image of a single posture, in its original form, (b) the same image with low-variance pixels masked out (c) the image again, reconstructed as an image, but using only the *L* PCA-generated features.

Eliminating relatively static candidate features in order to focus analysis on only those candidate features conceivably related to vocal tract action still leaves an unwieldy number of features (68^2^/4 = 1156) to consider. Thus, Principal Component Analysis (PCA) was employed to further reduce the data dimensionality. *Z* = *YC*_*L*_ was computed, where *C* is the matrix whose columns are eigenvectors of *YY*^*T*^, and *C*_*L*_ is a matrix containing only *L* columns that represent eigenvectors with the highest eigenvalues (i.e., the largest principal components). The value *L* was chosen so as to retain ≥ 85% of the total variance among all features for each subject being analyzed. This level of residual variance was achieved with *L* was approximately equal to 50. The resulting matrix *Z*, of size *P* by *L*, was used for all subsequent analyses. The matrix *Z* contained a relatively low-dimensional representation of the vocal tract configuration captured by rtMRI at a time nearest to an articulatory target. Images illustrating the key stages in this image pre-processing pipeline are shown in Fig 6. It is worth noting that performing PCA with the correlation matrix, rather than with the covariance matrix, as is typical, might provide an alternative method of dimensionality reduction that obviates the need for image intensity correction.

Syllabification was performed based on the forced alignment results, beginning with the word-level transcription from the adaptive forced alignment procedure. Words were translated into phoneme sequences finding their entries in the CMU Pronouncing Dictionary, which are already syllabified. Syllables were subsequently divided into onset, nucleus and coda by identifying the vowel as the nucleus, and considering all phones preceding the nucleus as part of the onset, and all phones following the nucleus as part of the coda. This syllabification allowed for (a) partitioning tasks into the meaningful categories of interest with respect to syllable structure, and (b) calculation of syllable position-specific movement times.

### Distance & width calculations

The vector *F*^*g*^ is an articulatory configuration which represents the present operational definition of the articulatory target associated with a given phoneme *g*. Note the adoption here of notation that indicates vector indices as superscripts. This notation occurs throughout the present section, and should not be confused with the use of superscripts as exponents. The text indicates when a given variable represents an index. The specific phoneme whose target is represented by *F*^*g*^ is indexed by *g*, which is a numerical index from 1 to 35 that uniquely specifies an American English phoneme. The vector *F*^*g*^ is defined as the mean configuration vector associated with the phoneme indexed by *g*:

$$\begin{array}{c} F^{g}=\frac{1^{T}diag\left(S_{g}\right)Z}{\|S_{g}{\|}_{1}} \end{array}$$ (9)

where **1** is a vector of ones. A phoneme vector *Π* of length *P* whose *p*^*th*^ element is *Π*^*p*^. Elements of *Π* are numerical American English phoneme indices from 1 to 35, just like *g*, and representing the phoneme associated with row *p* of *Z*. The vector *S*_*g*_ is also of length *P*, and $S_{g}^{p}=1$ whenever Π^*p*^ = *g*, and is 0 elsewhere. Therefore, the numerator of Eq 9 defines a sum taken over all rows of *Z* for which *S*_*g*_ = 1 (i.e., a sum over all vectors representing examples of articulatory targets associated with a specific phoneme).

For every pair of phoneme indices *g* and *h*, the distance between them, used in the calculation of Fitts law in the present analysis, is *D*_*gh*_. The value of *D*_*gh*_ is taken to be the Euclidean spatial distance between the associated phoneme targets in *L*-dimensional articulatory space. Specifically, the distance *D*_*gh*_ = ∥*F*_*g*_ − *F*_*h*_∥. A graphical representation of this quantity can be seen in Fig 4.

For every pair of phoneme indices, the time taken to reach phoneme *h* from phoneme *g*, used in the calculation of Fitts law in the present analysis, is *T*_*gh*_ (that is, *MT* in Fitts’ original formulation). The value of *T*_*gh*_ is taken to be the mean time between the ordered phoneme pairing indexed by *g* followed by *h* across all instances of that sequence:

$$\begin{array}{c} T_{gh}=\frac{1^{T}diag\left(S_{gh}\right)\Gamma -1^{T}diag\left(S_{hg}\right)\Gamma }{\|S_{g}{\|}_{1}} \end{array}$$ (10)

where *S*_*gh*_ is a vector that is 1 whenever Π_*p*_ = *h* and Π_*p*−1_ = *g*. Similarly, the vector *S*_*hg*_ is 1 whenever both Π_*p*_ = *g* and Π_*p*+1_ = *h*. *T*_*gh*_ was calculated separately for each of the five syllable position-based categories (listed above) to facilitate further analysis with an eye toward the hypothesized importance of syllable position in Fitts’ law-type relationships. This category-specific value for *T*_*gh*_ that was used on a category-by-category basis in the results discussed throughout the remainder of the present paper.

For a given phoneme and associated articulatory target, one could imagine several possible definitions for the width of that target. Looking outside the domain of speech production does not provide any clarification regarding this definitional question either, because there are several competing views on this key variable in the literature surrounding Fitts’ law. Fitts’ original experiments drew hard physical boundaries around the targets. Although physical boundaries (e.g., the hard palate) are relevant for certain speech actions (e.g., alveolar stops), there is not a clear mapping from the kinds of boundaries that Fitts utilized to the passive structures of the vocal tract. Measure of “effective” target width have also been explored, as expressed in terms of variability around the target [38, 39]. Additional definitions have been suggested that are based on the degree of overshoot (or undershoot) observed in approaching a target [13]. The present analysis considers an alternative definition that attempts to account for aspects of motor control that are unique to speech. Namely, due to the categorical nature of phonemic contrasts, the extent of the boundary around a given phoneme tends to be a function of the density of other phonemes near the given one.

This fact allows for the possibility of a width definition for speech tasks that is based on the density of targets in articulatory space. Consider the distance values *D*_*fh*_ for a given *h* and all *f* = 1, …, 35. These distance values with respect to *h* can be sorted and ranked, and—given a parameter *k*—one can select the distance between *F*_*h*_ and the *k*^*th*^ closest vector $F_{f_{k}}$. That distance can be used as the basis for a high-dimensional k-nearest-neighbor density calculation. The probability density of configuration vectors in the neighborhood of *F*_*h*_ will be:

$$\begin{array}{c} Q_{h}=\frac{k}{35\frac{\pi^{L/2}}{\Gamma (\frac{L}{2}+1)}D_{f_{k}h}^{L}} \end{array}$$ (11)

where Γ(*x*) is the gamma function and 35 is the number of phonemes under consideration (24 consonants and 11 vowels, with no diphthongs or rhoticized vowels). The width can be calculated from this probability density as *W*_*g*_ = −log_2_(*Q*_*g*_).

**Fig 7:**
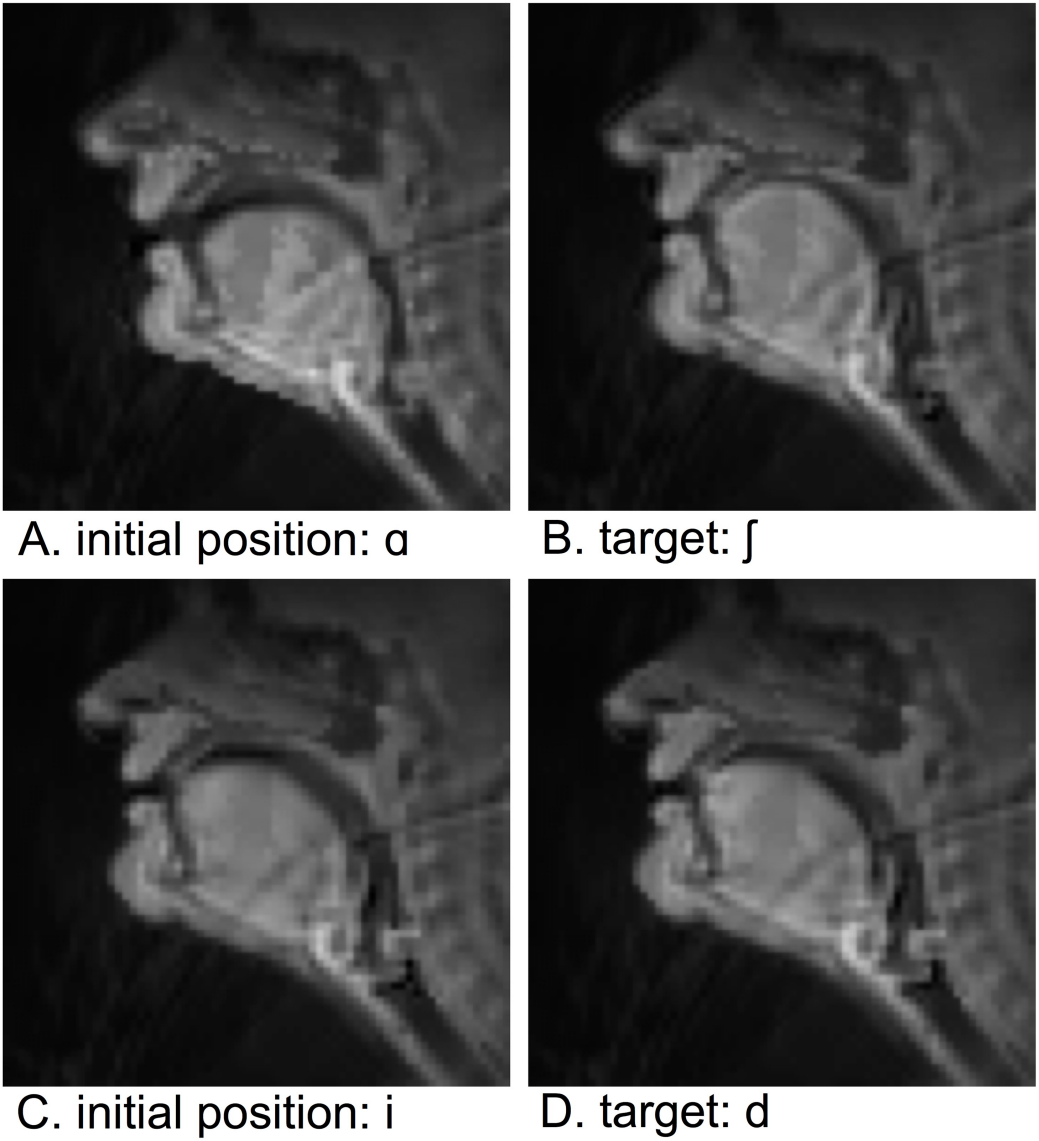
Example high- and low-ID tasks for subject M2. The top row, (a)-(b), represents one of the highest ID tasks, while the bottom row (c)-(d) represents one of the lowest. Images were reconstructed from the *L* articulatory features in *Z* (see text).

Given definition for *D*_*gh*_, *T*_*gh*_ and *W*_*h*_, for any phoneme indexed by *h* and presented in the context of another phoneme *g*, it is possible to apply Fitts’ law over the present data using Eq 1. In particular, one can calculate *ID* as *ID*_*gh*_ = log_2_(2*D*_*gh*_/*W*_*h*_). Subsequently, one can look for linear relationships between *ID* and *T*_*gh*_. By Eq 2, one would expect that *T*_*gh*_ = *a* ⋅ *ID*_*gh*_+ *b*, for some coefficients *a* and *b*. Images of initial positions and targets for one high-ID example and one low-ID example are shown in Fig 7.

### Metric

The strength of the relationship between MT and ID was assessed using linear correlation (Pearson’s *r*), in keeping with the linear form of Fitts’ law. The theoretical considerations discussed above imply that speed-accuracy tradeoffs should conform to Fitts’ law, and moreover that a true Fitts’-type relationship between MT and ID should be strictly linear. The present analysis, and associated choice of metric, is therefore oriented toward quantifying the degree of linear relationships in the data, if any exist. Any nonlinear relationship would not conform to Fitts’ law, and therefore would not be of interest presently. Pearson’s *r* has been widely used in the literature for assessing Fitts’ law-type relationships (see, e.g., [40]), presumably for this reason. Note that, even though Pearson’s correlation coefficient is an appropriate metric for the type of relationships sought in this study, a violation of certain assumptions—primarily normality, homoscedasticity and lack of outliers—may influence the number of type I and type II errors incurred in testing for significance of the specific correlation coefficients observed. These key assumptions were checked graphically by inspection of the ID versus MT scatter plots, and no reason was found to further question their validity in the present context.

## Results and discussion

The correlation coefficients calculated between ID and MT are shown in Table 1, divided by syllable position-specific category and by subject. Performing this analysis separately for each subject, and once for each task category, means that a total of 5 × 10 = 50 individual correlations were calculated, with 50 corresponding tests for statistical significance. It therefore became necessary to consider the significance of these results in light of some kind of multiple comparisons adjustment. It is not clear whether or how much these individual correlations are dependent, so statistical significance of the correlation coefficients is shown at three distinct threshold values: *α* = 0.05 (Fisher’s traditional value), *α* = 0.01 (an intermediate value) and *α* = 0.001, which is the conservative, Bonferroni-adjusted threshold value.

**Table 1:** Pearson’s r (first line of each cell) between movement time (MT) and index of difficulty (ID) for all subjects, divided by syllable position-specific category. Significance at the *α* = 0.05, *α* = 0.01 and *α* = 0.001 level are indicated by *, ** and ***, respectively. The 95% confidence intervals are indicated in square brackets on the second line of each cell. The p-values associated with each correlation coefficient are listed on the third line of each cell, along with the associated number of data points in parentheses.

	Onset—Nucleus	Nucleus—Coda	Onset—Onset	Coda—Coda	Coda—Onset
M1	r = -0.06	r = 0.21*	r = -0.01	r = -0.08	r = 0.05
	[-0.22, 0.11]	[0.00, 0.39]	[-0.37, 0.36]	[-0.46, 0.33]	[-0.07, 0.17]
	0.48 (141)	<0.05 (92)	0.96 (29)	0.72 (25)	0.40 (266)
M2	r = 0.49***	r = 0.72***	r = -0.13	r = 0.52***	r = 0.37***
	[0.37, 0.59]	[0.62, 0.79]	[-0.45, 0.23]	[0.25, 0.72]	[0.28, 0.46]
	<0.001 (183)	<0.001 (127)	0.48 (33)	<0.001 (40)	<0.001 (384)
M3	r = 0.03	r = 0.29***	r = -0.25	r = -0.28	r = 0.05
	[-0.12, 0.17]	[0.12, 0.45]	[-0.55, 0.11]	[-0.54, 0.03]	[-0.05, 0.14]
	0.70 (187)	<0.001 (125)	0.18 (32)	0.07 (41)	0.34 (411)
M4	r = 0.07	r = 0.33***	r = -0.35*	r = -0.30	r = 0.15**
	[-0.07, 0.22]	[0.16, 0.48]	[-0.62, -0.00]	[-0.56, -0.00]	[0.05, 0.24]
	0.32 (184)	<0.001 (126)	<0.05 (33)	0.05 (42)	<0.01 (413)
M5	r = -0.01	r = 0.38***	r = -0.21	r = 0.18	r = 0.03
	[-0.17, 0.15]	[0.19, 0.54]	[-0.53, 0.16]	[-0.22, 0.53]	[-0.08, 0.14]
	0.89 (147)	<0.001 (97)	0.26 (30)	0.38 (26)	0.56 (312)
F1	r = 0.36**	r = 0.41***	r = 0.02	r = 0.46**	r = 0.24***
	[0.23, 0.48]	[0.26, 0.55]	[-0.33, 0.37]	[0.18, 0.67]	[0.14, 0.33]
	<0.01 (183)	<0.001 (126)	0.91 (32)	<0.01 (41)	<0.001 (389)
F2	r = 0.30***	r = 0.49***	r = 0.40**	r = 0.38*	r = 0.17***
	[0.16, 0.43]	[0.35, 0.61]	[0.07, 0.66]	[0.08, 0.62]	[0.07, 0.27]
	<0.001 (182)	<0.001 (127)	<0.01 (33)	<0.05 (41)	<0.001 (388)
F3	r = -0.04	r = 0.30***	r = 0.20	r = -0.33*	r = 0.05
	[-0.19, 0.10]	[0.13, 0.45]	[-0.16, 0.52]	[-0.58, -0.03]	[-0.05, 0.14]
	0.56 (182)	<0.001 (126)	0.27 (32)	<0.05 (41)	0.34 (413)
F4	r = -0.11	r = 0.31**	r = -0.26	r = -0.18	r = -0.03
	[-0.26, 0.05]	[0.12, 0.48]	[-0.56, 0.11]	[-0.51, 0.21]	[-0.15, 0.08]
	0.19 (153)	<0.01 (102)	0.17 (30)	0.37 (28)	0.60 (291)
F5	r = 0.06	r = 0.28**	r = -0.12	r = -0.04	r = 0.06
	[-0.09, 0.20]	[0.11, 0.43]	[-0.45, 0.23]	[-0.35, 0.27]	[-0.04, 0.15]
	0.41 (184)	<0.01 (126)	0.50 (32)	0.80 (40)	0.26 (407)

**Fig 8:**
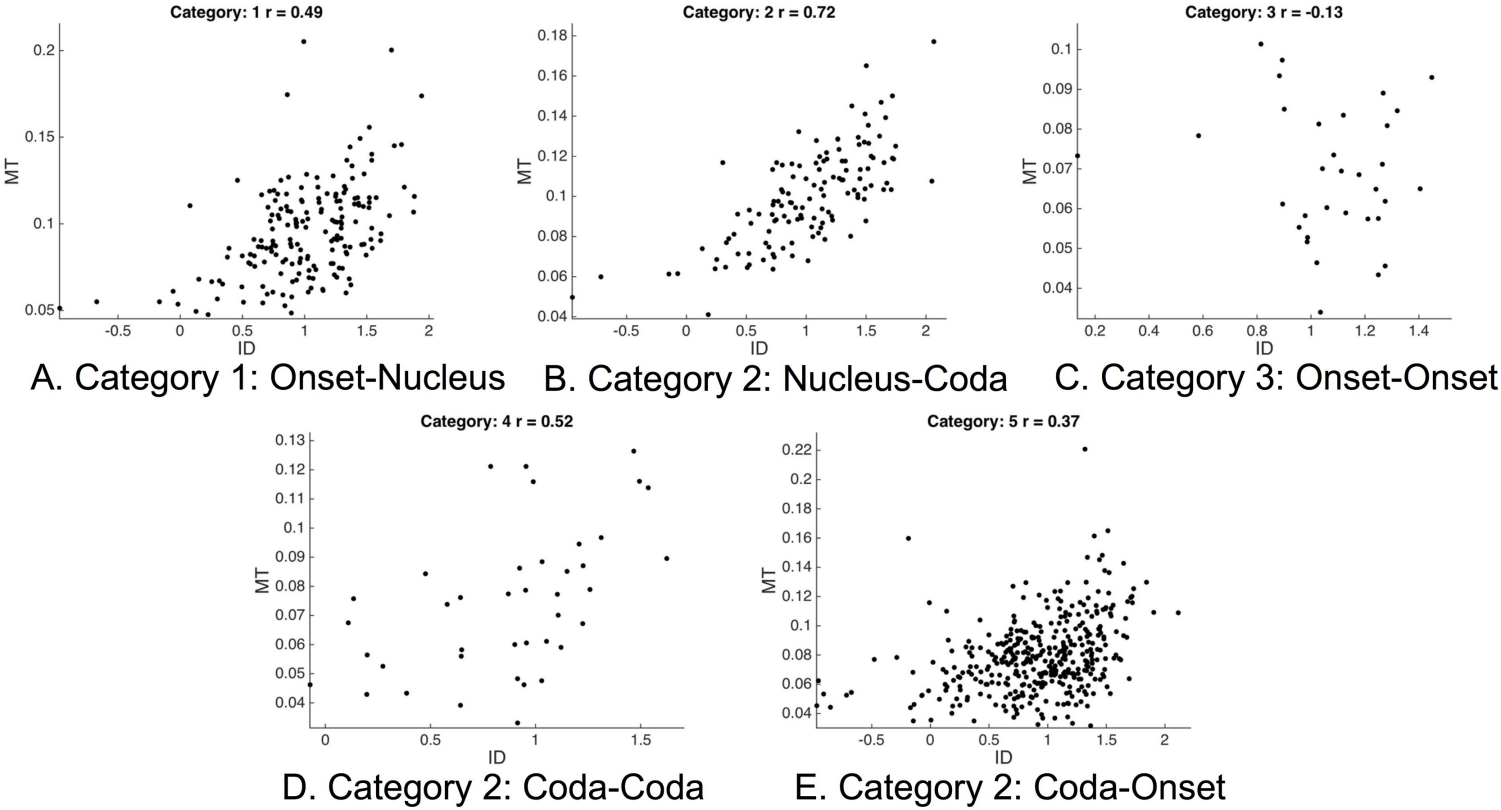
Movement time (MT) vs. index of difficulty (ID) for subject M2. All context-target tasks are shown, divided by syllable position-based category (see text for details concerning categories).

Subject M2 had the generally highest correlation values of all subjects, indicating that the Fitts-style relationships were strongest and clearest for that subject. Fig 8 shows MT versus ID for subject M2, showing the strength and nature of those relationships for each of the syllable position-specific task categories. The correlation values corresponding to each category, and the associated p-values, are shown above each plot.

### Articulatory difficulty

Results suggest that the difficulty associated with targeted articulatory kinematics is highly variable in speech production. ID ranges from approximately 0.25 to 1.75 bits for all subjects. A few general patterns in the distribution of ID can be noted. Difficulty was assessed by looking at the overall average ID associated with a given target position. ID values for each subject were normalized between 0 and 1, in advance of taking the mean ID for each task across subjects. The mean ID was then calculated for each task with a consonant target, given a vowel initial position. These tasks, listed from most difficult to least difficult, were: ʒ, ʧ, θ, h, ∫, p, w, ʤ, ð, b, g, k, j, f, ŋ, v, z, s, m, l, d, r, n, t. The mean ID was also calculated for each task with a vowel target and a consonant initial position. These tasks, listed from most difficult to least difficult: ʊ, o, ɑ, ɔ, e, æ, u, ε, i, i, ə,. Fig 7 shows example low- and high-ID tasks for subject M2.

Consonant tasks involving labial articulation, whether primary (/p/, /w/, /b/), or secondary (/∫/), tend to have a higher difficulty. Nasals and liquids all ranked as lower difficulty. Fricatives /∫/, /θ/, /ð/ and affricates show higher ID, a fact that is consistent with Hardcastle’s assertion that fricatives—and perhaps by extension, affricates—are the sounds of speech requiring the greatest accuracy. This does not necessarily include all fricatives, however, as /z/ and /s/ were ranked relatively lower. The difficulty associated with producing fricative and affricates is particularly evident when examining them in the context of low, back vowels, where the distance from the initial position to the target position is lengthened. One can also observe that these articulatory tasks require more time to complete than other tasks. Also consistent with Hardcastle is the observation that stop consonants—particularly alveolar—require little accuracy, and are therefore not difficult. Since distance is a factor under consideration, one can see that this effect is again emphasized when the initial position is a high, front lax vowel. It is important to remember that sibilants may have complex aerodynamic requirements, and off-midsagittal kinematic requirements, that will not necessarily show up in the purely midsagittal kinematic analysis in the present work.

Vowel targets that were low and back had a higher level of difficulty, as compared to the relatively lower difficulty of high and front vowels. Vowels that were not directly along this primary low-back/high-front axis, including the high-back vowel /u/ and the low-front vowel /æ/, appeared together toward the middle of the vowel difficulty ranking. Schwa was ranked as the least difficult vowel to produce. This ranking is consistent with the importance of D in computing ID. With a schwa target, the speech articulators should have, on average, a shorter distance to travel from other initial positions. It has been shown that, although it has a distinct phonetic identity, schwa is perceptually and articulatorily similar to “articulatory setting”, which is the neutral posture from which speech actions are deployed and to which they tend to return [41]. This neutral posture is hypothesized to be kinematically advantageous, just as there is evidence that it is mechanically advantageous [42]. Conversely, low-back vowels should require the speech articulators to travel longer distances from a variety of initial positions, in order to reach kinematic targets in the region of the pharynx.

One other issue of note concerns that fact that the distance and width parameters do not seem to contribute equally to the index of difficulty, given the present definitions, and current data. Although both parameters influence the final value of ID, difficulty seems to be determined to a much larger degree by distance than by width. For instance, for subject M2, the correlation between D and ID across all diphones is substantially greater (Spearman’s *ρ* = 0.987, *n* = 1190, *p* ≪ 0) than the correlation between W and ID (Spearman’s *ρ* = 0.222, *n* = 1190, *p* ≪ 0). Similar trends are seen across all subjects. Note that this correlation between W and ID is in the opposite direction from expected, based on the equation for ID. This may be due to the fact that, for these data, D and W seem to be positively correlated (e.g., for subject M2: Spearman’s *ρ* = 0.3565, *n* = 1190, *p* ≪ 0).

### Speed-accuracy tradeoff

Results suggest that targeted speech actions exhibit a clear tradeoff between speed and accuracy in certain task categories, and with substantial interspeaker variability. Significant correlations can be seen in the data that correspond to the relationship between MT and ID predicted by Fitts’ law. The strength of that relationship varies across speaker and task type. The strongest and most highly significant of such relationships are seen for Nucleus-Coda tasks across all subjects. Onset-Nucleus and Coda-Onset tasks also showed generally high correlations that were significant for at least three subjects (M2, F1 and F2, but also M4 for Coda-Onset tasks). Note that many fewer Onset-Onset and Coda-Coda tasks exist, as compared to other task types. Note also that, for non-significant correlations, the 95% confidence intervals presented in Table 1 may help in interpreting whether these low correlation coefficients represent the lack of evidence for speed-accuracy tradeoffs consistent with Fitts’ law, or instead represent evidence for the lack of such relationships. For instance, the consonant-consonant tasks (i.e., Onset-Onset and Coda-Coda) appear to have rather wide confidence intervals, whereas the other position-specific categories have much narrower confidence intervals. This may indicate a higher likelihood that Fitts’ law-type relationships are not present in Onset-Nucleus, Nucleus-Coda and Coda-Onset tasks, versus relative uncertainty on this point for consonant-consonant tasks, given the data.

For speakers that display significant correlations between *ID* and *MT*, appearance of associated scatter plots (see, e.g., Fig 8) suggest that the relationship between these two variables is roughly linear. This qualitative assertion is supported by the fact that nonlinear correlation techniques appear to judge the strength of the correlation as approximately the same. For example, taking the data from Subject M2 from the Nucleus-Coda condition (pictured in Fig 8), Spearman’s *ρ* has a value of 0.71. Abundant added noise is evident in the measured relationship. There is also a notable deviation from a purely linear relationship at small ID values, where MT appears to hit a minimum value around 50ms. This floor effect may reflect physiological constraints on the production apparatus. A similar “flattening” of the ID-MT relationship at small values of ID has been observed in other investigations of Fitts’ law and, in fact, the linearity of Fitts’ law has been called into question by several studies (e.g., [38, 43, 44]). It is important to emphasize that the true functional form of the underlying relationship between ID and MT, though an interesting consideration for future work, is not at stake in the current work. Based on the theoretical considerations outlined above, one should expect the speed-accuracy tradeoffs should conform to Fitts’ law, and a true Fitts’-type relationship between the variables of interest should be strictly linear. The present analysis is therefore oriented toward quantifying the degree of any specifically linear relationship, if it exists. Any nonlinear relationship would not be a Fitts’-type relationship, and therefore would not be of interest presently. Pearson’s *r* remains the best mathematical tool to address this, the primary question of interest.

As noted in a related, preliminary analysis by the present authors [18], the correlations observed in speech production tasks on the present data are relatively modest compared to those observed in other domains of human movement, where it is not uncommon to see correlation coefficients above 0.9 [45]. The highest correlation value observed on the present data was 0.72, as exhibited by subject M2 on the Nucleus-Coda task. Moreover, the magnitude of the correlation values also appears to be somewhat dependent on the specific task type (as hypothesized) and the specific subject under consideration. A key question raised by such results is why this seemingly fundamental tradeoff, that has been well-established in other motor domains, appears to be somewhat modestly and variably obeyed by speech motor tasks. Several possible explanations are considered in the present discussion.

The class of movements considered ballistic (i.e., occurring without feedback control while movement is underway) provide an example explanation to consider. It has been argued that ballistic movements, including eye saccades, do not obey Fitts’ law because a lack of feedback means that movement time does not depend on the required accuracy of performing the task, but only on the movement amplitude [6, 8]. It is possible that certain speech production actions implement ballistic control. However, even despite their rapidity, speech motor tasks are typically not modeled as being ballistic in nature. Major models of speech motor control involve feedback at fine temporal scales. As discussed earlier, the Task Dynamics model relies on feedback, and leads naturally to precisely the kind of speed-accuracy tradeoffs described by Fitts law [20]. Other prominent models of speech motor control, such as the DIVA model [46] and State Feedback Control [47], also rely on feedback, although the connection to Fitts’ law has not been explicitly made. Therefore, rather than concluding that each of these models is inaccurate, and that ballistic control of speech movements provides a better explanation of the present data, a more likely explanation for the weak and variable observed relationships is that the definition of speech tasks used in the present work needs to be revised.

One way to reconsider the presently-used definition of speech tasks is to make them multimodal, for instance by incorporating prosodic constraints. As mentioned above, speech has multiple levels in which accuracy may be demanded. Speech motor actions have communicative and prosodic goals, in addition to kinematic requirements. Temporal constraints likely exist as part of those goals, both at the level of phonetic segments (e.g., lengthening as a phonemic contrast) and suprasegmentally (e.g. accenting). Here, attention has been paid to the influence of the position of a speech task within the syllable. However, factors that wield influence over speech tasks may also vary as a function of other positional factors—e.g., the position of the syllable in the word, and the position of the syllable in the utterance. Stress and focus marking the syllable or the word containing the syllable may also influence speech timing, as well as speaking style and register, and even the neurocognitive state of the speaker (e.g., emotional, neurological). Indeed, the objective function for speech motor control might be formulated as an information-theoretic measure exemplifying both the achievement of the kinematic goal and any temporal information encoding, including active and incidental temporal aspects. A modification of speech tasks (and, perhaps, Fitt’s law itself) is needed to account for these various levels of task requirements, and associated timing requirements. A closer consideration of the above factors may provide fruitful directions for future investigations into Fitts’ law that can inform such modifications for speech production. Moreover, speech tasks should potentially also allow for contextually modified targets. Enhancing speech tasks in this way would also allow for a natural way to capture co-articulation in targets, as opposed to the fixed targets considered in this work.

Another important change to the presently-used definition of speech tasks may be to account for non-sequential, overlapping articulatory targets, as opposed to the purely sequential tasks considered in this work. In fact, the results already indicate the need for such an enhancement. It has been well established that speech articulatory gestures at certain positions in the syllable are highly overlapping, whereas others are more sequential [28–30]. Specifically, consonants within an onset would be expected to overlap with each other extensively, and would overlap with the succeeding nucleus, as well. The nucleus, in contrast, should overlap very little with the succeeding consonant in the coda. The present results clearly show that the correlations are strongest for the Nucleus-Coda tasks across all subjects, which is exactly what would be predicted by the sequential, non-overlapping nature of the gestures involved at that position in the syllable. The Onset-Onset tasks, for which the assumption of sequential targets may be inappropriate, show the poorest overall correlations. If the current definition of speech tasks is retained, a general relationship between gestural overlap and the presence of Fitts’ law-type effects in speech articulation should be expected. Relatedly, this interpretation implies certain predictions regarding the expected presence of Fitts’ law-type tradeoffs cross-linguistically. The degree of gestural overlap has been hypothesized to be language-specific to some extent, with some published evidence in support of that idea from English versus Russian [48], and from Georgian [49]. Future work could examine whether languages, like Russian, that may display relatively less gestural overlap, also obey Fitts’ law more closely.

There are substantial interspeaker differences in the strength of correlations between ID and MT. These differences are evident in the Nucleus-Coda tasks, where most subjects displayed significant correlations, but to different degrees. Interspeaker differences are also evident for other tasks, such as the Onset-Nucleus tasks, where some subjects showed marginal correlations (e.g., M3 and F5) and others (e.g., M2 and F2) showed highly significant correlations. Important questions remain regarding an explanation for this prevalent interspeaker variability. These differences may reflect interspeaker differences in control strategies, that in turn are a function of speaking rate, age, social community, morphological (i.e., physical) variation, and a variety of other factors. Morphological variation, being by definition a fundamental influence on kinematics, holds potential as an explanation for interspeaker differences even in a seemingly fundamental law of motor control and behavior like Fitts’ law. It is known that speakers vary widely in terms of a number of morphological characteristics, including vocal tract length [50, 51] and relative proportions [52, 53], as well as hard palate and posterior pharyngeal wall shape [54], and many other parameters. There is growing evidence that differences in morphology of the speech apparatus all influence the production of specific speech sounds at the level of articulatory goals and kinematics [55–58]. A particularly intuitive example comes from indications that individuals vary in terms of their tongue size relative to the size of the entire speech apparatus [59]. It seems reasonable to expect that a smaller relative tongue size will result in longer articulatory distances travelled within the oral and pharyngeal cavities, on average, resulting in a wide range of values for ID. This wider range of ID might, in turn, cause the relationship between ID and MT to stand out against any noise in the data. As another example, it has already been discussed in the present work how low-back vowels appear to be the most difficult vowels to produce, and it was suggested that this may be the result of longer articulatory distances associated with producing them. There may be complex interactions between this observation and morphological variation in the relative length of the pharyngeal cavity [60], and perhaps its volume. The potential connection between vocal tract morphology and Fitts’ law for speech production merits further attention.

It should be noted that the use of rtMRI adds several sources of variability to the present analysis that may impact the resulting correlation values, and even limit the generality of the present results. The temporal resolution of rtMRI may limit the accuracy of determining a reconstructed video frame nearest to the temporal center of a given phone. Recent advances in rtMRI may alleviate this limitation, as rtMRI is a currently-evolving technology [61, 62]. The quality of forced phoneme alignment will also limit the accuracy of determining this same variable. If speaking rate is a factor, then the correlation values from Table 1 should, in turn, be correlated with speaking rate. Speaking rate was computed for all subjects by looking at the mean time between adjacent syllable nuclei to get an estimate of syllable rate. Pearson’s correlations were found between these values and the correlation values for Nucleus–Coda Consonant (r = -0.64, p = 0.047) and Onset Consonant–Nucleus (r = -0.63, p = 0.049). The fact that speaking rate is a factor indicates that the current data may have frame rates that are at the boundary of usefulness for the analysis done in this study. Higher frame rates would be preferable in future work. Additional variability may stem from non-Gaussian noise in reconstructed rtMRI images that is expected to be present in pixel intensity values. Added variability in the data and analysis would have the clearest impact on the Onset-Onset and Coda-Coda task results, due to their much smaller number. Data are also limited to a midsagittal view of the speech articulators, meaning not all kinematic aspects are captured in the data.

Although the purpose of the present paper was to provide a fundamental investigation into whether speech articulatory kinematics conform to Fitts’ law, it is possible that knowledge of articulatory difficulty and its relationship with movement time in speech may have practical applications, as well. For speech phenomena that obey Fitts’ law, observed changes in difficulty or movement time each imply changes in the other, and perhaps the extent to which changes in one variable can be attributed to changes in the other. This becomes relevant, for example, when considering that decline in speaking rate—perhaps as a result of increases in movement time—is associated with various kinds of neurological decline [63, 64]. Decreases in speaking rate may represent a compensatory mechanism in order to maintain accuracy as difficulty increases. Relatedly, but conversely, it has been demonstrated that accuracy and intelligibility decline at markedly increased speaking rates in normal speakers [65, 66]. Articulatory difficulty may also be useful for explaining certain speech phenomena on its own. For instance, task- and phoneme-specific variation in difficulty may help to explain why fricatives tend to be acquired later than stops [67], and why some productions are more quickly impacted when the condition of the motor system changes. Sleepiness and alcohol intoxication lead to the salient changes in fricative production associated with so-called “slurred” speech [68, 69]. There would appear to be opportunities for explaining together changes in speed, accuracy, difficulty and movement time during speech acquisition and speech pathology that merit further investigation.

## Conclusion

This paper has presented an analysis of speech articulation from a large database of real-time magnetic resonance (rtMRI) data, in order to assess whether articulatory kinematics conform to Fitts’ law. It appears that certain aspects of speech production do conform to Fitts’ law, while the strength of that relationship varies across speaker and context-target type. The strongest such relationships are seen for VC context-target tasks, with CV tasks showing nearly as strong correlations. Also presented was a novel methodology for addressing the challenges inherent in performing Fitts-style analysis on rtMRI data of speech production, from defining the key quantities to extracting them from rtMRI data. Finally, a novel mathematical argument was presented for the expectation of Fitts’ law in speech production, and why one expects to observe behavior consistent with the law on the basis of Task Dynamics and the VITE neural model of directed movement. Future work should focus on addressing the remaining methodological challenges. Among these challenges are higher frame rate data, and exploring additional definitions of the key relevant quantities.
